# Associations of Indoor Nighttime Ventilation with the Relative Abundances of Typical Pathogenic Bacteria and Fungi in Settled Dusts from Floor, Desk, and Bed of University Dormitories

**DOI:** 10.3390/microorganisms14071521

**Published:** 2026-07-12

**Authors:** Wei Liu, Wangjin Lai, Yu Zhang, Jinze Du, Ying Chen, Zhi Li, Nan Zhang, Jiao Cai

**Affiliations:** 1 School of Civil and Hydraulic Engineering, Chongqing University of Science and Technology, Chongqing 401331, China; 2 Institute for Health and Environment, Chongqing University of Science and Technology, Chongqing 401331, China; 3 Beijing Key Laboratory of Green Built Environment and Energy Efficient Technology, Beijing University of Technology, Beijing 100124, China; 4 Chongqing Research Institute of Beijing University of Technology, Chongqing 401100, China

**Keywords:** university dormitories, cumulative ventilation, settled dust, pathogens, indoor microbial exposure

## Abstract

Inadequate ventilation in university dormitories risks microbial exposure, but its association with pathogen prevalence in dust across indoor surfaces remains unclear. In this study, we monitored nocturnal air exchange rates (AERs) over a year in dormitory rooms and collected settled dust samples from beds, desks, and floors across four seasons. Based on the World Health Organization (WHO) priority bacterial and fungal pathogen lists, 34 pathogenic species were initially identified from gene sequencing data. We built multivariate regression models to evaluate the associations of daily cumulative ventilation status before dust sampling with the distribution of these pathogens. The results indicated a low compliance rate (AER ≥ 0.7 h^−1^, 43.2–52.1%) for ventilation in the dormitories. *Fusarium* and *Acremonium* were detected in most samples (92.3–99.5%). The modulatory effect of nighttime cumulative ventilation varied heavily across pathogen species and surface types. The relative abundance of *Candida tropicalis* on bed dust exhibited a stable negative correlation with long-term cumulative AER (β: −0.42 to −0.50), whereas the relative abundance of *Salmonella* correlated positively with higher AERs. This study demonstrates that ventilation is not a universal pathogen-control measure, revealing its temporal cumulative characteristics and spatial heterogeneity of ventilation in modulating indoor pathogens, and provides a theoretical foundation for implementing precision ventilation strategies in university dormitory environments.

## 1. Introduction

People now spend approximately 90% of their time indoors, directly linking indoor environment quality to public health outcomes [1,2,3]. Among various indoor pollutants, bioaerosols, encompassing bacteria and fungi, have garnered significant attention due to their potential pathogenicity [4,5,6]. Substantial epidemiological evidence links exposure to specific indoor microbes with adverse health outcomes, such as allergic reactions, asthma, and other immune-regulated disorders [1,7,8,9,10,11]. To address these threats, the World Health Organization (WHO) published the WHO Fungal Priority Pathogens List (2022) [12] and the WHO Bacterial Priority Pathogens List (2024) [13], establishing an authoritative framework for monitoring high-risk pathogens.

University dormitories, characterized by confined spaces and high occupant density, serve as primary residential settings where students spend over 10 h daily [14,15]. Consequently, dormitory environmental quality poses a continuous and direct health threat to occupants [16,17,18]. Ventilation is a key measure for regulating indoor environments and reducing the risks associated with airborne pollutants and microbial exposure [19,20,21,22,23]. Research demonstrates that higher ventilation rates effectively mitigate the infection risk of airborne pathogens [24,25], and in certain contexts, their role may even surpass that of exposure duration and occupant density [26]. However, unlike residential apartments equipped with mechanical ventilation systems, university dormitories primarily rely on window operations and door infiltration, resulting in generally low overall ventilation efficiency [27]. Field measurements consistently show low air exchange rates (AERs) in university dormitories across multiple regions, frequently falling below international standards [28,29,30,31].

Existing studies have identified inadequate ventilation as a critical factor contributing to indoor microbial pollution [32,33,34,35]. In university dormitories, this widespread inadequate ventilation not only correlates with higher respiratory infection rates [36,37,38,39] but also continuously drives the accumulation of airborne pathogens [40,41,42,43]. Despite these recognized links, a critical research gap remains: the modulatory effect of ventilation on indoor microbiomes is rarely instantaneous. Most existing research relies on single-day or snapshot ventilation measurements [22,44,45,46], largely neglecting the temporal accumulation process of indoor pathogens. While many studies focus on airborne microbes, bioaerosols are highly transient and fluctuate rapidly due to immediate environmental disturbances [47]. Conversely, settled dust acts as a continuous environmental sink that integrates microbial accumulation over time, providing a more stable and reliable indicator of long-term occupant exposure [1,48,49].

This study introduces the novel metric of “cumulative nighttime ventilation.” Based on longitudinal field monitoring data, we investigate the associations of daily cumulative ventilation, defined as the average AER over the 1–7 days preceding dust sampling, with typical pathogens present in settled dust from various locations within university dormitory, and aim to provide a theoretical foundation for developing targeted ventilation control strategies in the indoor environments as university dormitory.

## 2. Methods

### 2.1. Study Sites and Sampling

**Figure 1:**
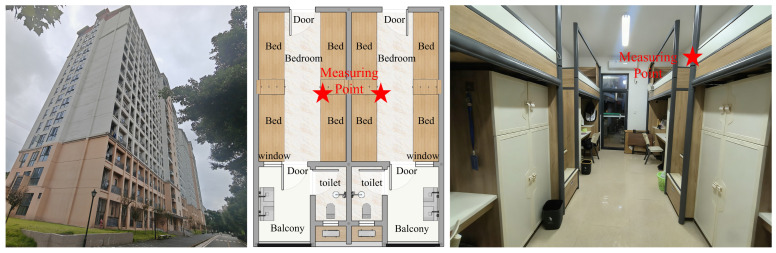
Schematic diagram of the university dormitories and measuring point distribution.

This study was conducted in a 15-story university dormitory building (occupied since 2022) located in Chongqing, China. To account for spatial and orientation variations, a total of 12 dormitory rooms were selected from the 8th floor (6 rooms, 24 male students) and the 12th floor (6 rooms, 24 female students), with each floor comprising three south-facing and three north-facing rooms. All rooms housed four students and featured a standardized layout with lofted beds above study desks. Ventilation was dependent on the opening of balcony doors and windows (Figure 1).

Settled dust sampling was conducted in each season from 1 May 2024 to 30 April 2025. Sampling dates were selected based on the predominant weather pattern for each season and further calibrated to ensure that the daily temperature was within ±2 °C of the seasonal average, humidity fell within the typical range for that weather type, and no extreme weather events had occurred in the 72 h preceding sampling. Dust samples were collected from beds, desks, and floors in each dormitory using a 700 W household vacuum cleaner (Yangzi YZ-SC-X2; Ningbo Jiequ Electric Appliance Co., Ltd., Ningbo, Zhejiang, China) fitted with a custom stainless-steel nozzle, with each sample collected in about a 5 min period. To ensure methodological consistency, the sampling area for each site was strictly defined and kept identical across all longitudinal sampling campaigns. Specifically, the entire surface of the bed (0.9 m × 2.0 m) was vacuumed due to the relatively sparse dust accumulation, while a fixed area of at least 1 m^2^ was sampled for both the desk and the floor. The dust was collected on DR-85 circular filter media (85 mm diameter, 95% capture efficiency for particles ≥ 0.3 μm; Ningbo Shengbao Safe Technology Co., Ltd., Ningbo, Zhejiang, China). Following collection, filter membranes were wrapped in aluminum foil, sealed in bags, and stored at −80 °C until DNA extraction for high-throughput gene sequencing.

During each sampling, we administered questionnaires on dormitory environment characteristics and student lifestyle habits. The lifestyle habits included the frequency of airing and changing bed linens, bed-making habit after waking, desk cleaning frequency, and floor sweeping and mopping frequency. The detailed statistical characteristics of these lifestyle habits are summarized in Supplementary Table S1. Ultimately, a total of 552 valid settled dust samples were successfully collected across the four seasons. These comprised 142 samples from spring, 141 from summer, 136 from autumn, and 133 from winter. Regarding the specific sampling sites, the dataset included 178 samples from beds, 187 from desks, and 187 from floors. These samples were subjected to subsequent high-throughput sequencing for bacterial and fungal community analyses.

### 2.2. Indoor Ventilation Evaluation

Indoor temperature, relative humidity, and CO_2_ concentration were monitored using QD-G3 air quality sensors (Beijing Green Built Environment Technology Co., Ltd., Beijing, China). The measurement ranges and accuracies for each parameter were as follows: temperature 0–60 °C (±0.5 °C), relative humidity 0–99% (±10%), and CO_2_ concentration 0–5000 ppm (±70 ppm). The sampling interval for all parameters was set to 1 min. Before deployment, we cross-calibrated all sensors simultaneously for 12 h. One sensor was positioned in the center of each dormitory room at a height of 1.8 m above the floor to minimize interference from occupant activities (Figure 1). The air exchange rate (AER) was estimated using the CO_2_ concentration step-up method [50] based on the integral mass balance equation. Assuming well-mixed indoor air, the dynamic change in indoor CO_2_ concentration is expressed as $C_{t}=C_{out}+\frac{E}{V\cdot AER}+\left(C_{0}-C_{out}-\frac{E}{V\cdot AER}\right)\cdot e^{-AER\cdot t}$, where $C_{t}$ is the indoor CO_2_ concentration at time t, $C_{0}$ is the initial concentration, $C_{out}$ is the outdoor background concentration, E is the total CO_2_ emission rate of the occupants, and V is the room volume. We applied a non-linear curve-fitting method to iteratively solve for the AER that minimized the sum of squared errors between the theoretically predicted and actual continuous sensor readings. A detailed step-by-step example of this calculation, including the determination of occupant-specific emission rates, is provided in Section S1 of the Supplementary Materials. To avoid potential disturbances from frequent daytime activities, the nighttime period (22:00 to 08:00 in the next day) was selected for AER calculation, utilizing the rising segment of the CO_2_ concentration data. This selection was driven by both the methodological requirements of the tracer gas technique and the unique occupancy patterns of graduate student dormitories. During the daytime, graduate students primarily work in laboratories, leaving the dormitories largely unoccupied. This absence of a stable indoor CO_2_ generation source makes daytime AER estimation unreliable. Furthermore, frequent daytime door-opening events introduce significant interference. In contrast, during the nighttime (sleeping hours), dormitories are fully occupied, providing a continuous and stable CO_2_ emission source suitable for the step-up method. More importantly, this continuous nighttime occupancy represents the absolute primary period of residents’ prolonged exposure to the dormitory indoor environment. Furthermore, annual temperature and humidity data from all dormitories were exported, and the daily average values for each parameter were computed for each room to serve as the basis for subsequent data analysis.

Approximately 6% of the raw continuous monitoring data for AER, indoor air temperature, and relative humidity were missing due to sporadic sensor disconnections. These missing values were handled using multiple imputation. Five complete datasets were generated via the Markov Chain Monte Carlo algorithm, with the imputation model incorporating the continuous distribution characteristics of the variables and constraints based on their physical meanings to ensure result plausibility.

All subsequent statistical analyses were performed using the imputed datasets and focused on the indicator “Cumulative AER for *n* days before sampling” (where *n* = 1 to 7). This indicator is specifically defined as the arithmetic mean of the nocturnal AER over the *n* consecutive days immediately preceding the sampling day (e.g., the 3 days pre-sampling AER represents the average of AER from days 1, 2, and 3 before sampling). It was designed to reflect the cumulative ventilation level over different durations (1 to 7 days) prior to sampling, thereby assessing the sustained effect of dormitory ventilation rather than its instantaneous state on a single day. The overall ventilation compliance rates in different seasons were also analyzed according to the compliance threshold for residential ventilation (AER ≥ 0.7 h^−1^) recommended by the Standard for Indoor Air Quality (GB/T 18883-2022) [51].

### 2.3. Measurement of Bacteria and Fungi

Gene sequencing for this study was performed by Shanghai Majorbio Bio-pharm Technology Co., Ltd., Shanghai, China. Each collected dust sample was divided into two aliquots, designated for bacterial and fungal gene sequencing analysis, respectively. Genomic DNA was extracted from the dust samples using the DNeasy^®^ PowerSoil^®^ Pro Kit (QIAGEN, Germantown, MD, USA). The quality of the extracted DNA was assessed via 1% agarose gel electrophoresis and a NanoDrop2000 spectrophotometer. The targeted sequencing regions were the V3–V4 hypervariable region of the bacterial 16S rRNA gene and the ITS1 region of the fungal ITS gene. The primer pairs were 338F (5′-ACTCCTACGGGAGGCAGCAG-3′) and 806R (5′-GGACTACHVGGGTWTCTAAT-3′) for bacteria [52] and ITS1F (5′-CTTGGTCATTTAGAGGAAGTAA-3′) and ITS2R (5′-GCTGCGTTCTTCATCGATGC-3′) for fungi [53]. The PCR products were extracted from 2% agarose gels, purified using a PCR Clean-Up Kit (Yuhua, Shanghai, China), and quantified with a Qubit 4.0 fluorometer (Thermo Fisher Scientific, Waltham, MA, USA). Sequencing libraries were constructed using the NEXTFLEX Rapid DNA-Seq Kit and subsequently sequenced on the Illumina PE300/PE250 platform (Majorbio Bio-Pharm Technology Co., Ltd., Shanghai, China). Following sequencing, the raw paired-end reads were subjected to rigorous quality control. The sequences were demultiplexed, quality-filtered using FASTP to remove low-quality reads, and merged using FLASH. Taxonomic assignment of the high-quality sequences was performed using a Naïve-Bayes consensus taxonomy classifier, referenced against the SILVA database for bacterial 16S rRNA genes and the UNITE database for fungal ITS sequences.

Based on the gene sequencing results, pathogens within the dust samples were screened against the World Health Organization (WHO) Fungal Priority Pathogens List (2022) [12] and the Bacterial Priority Pathogens List (2024) [13]. The initial screening identified six pathogenic bacterial and 13 pathogenic fungal taxa. To ensure the robustness of subsequent statistical analyses, species detected only in very few samples (i.e., pathogens with an exceedingly low detection rate) were excluded. Thus, we retained three pathogenic bacterial taxa and seven pathogenic fungal taxa for in-depth analyses.

### 2.4. Statistical Analyses

We used a hierarchical statistical approach to evaluate the associations of cumulative ventilation with pathogens. First, we applied the Mann–Whitney *U* test to compare the distribution differences in pathogen relative abundance among the different sampling sites (bed, desk, floor). Then, we built multivariate logistic regression models to investigate the associations of cumulative ventilation (represented by the average AER over 1 to 7 days before sampling) with pathogen detection status (i.e., detected vs. non-detected). The generalized linear models (GLMs) were built to evaluate the associations of cumulative ventilation with the relative abundances of the 10 pathogens, respectively. Separate models were built for each target pathogens. All models considered season, dormitory orientation, indoor air temperature, and relative humidity as covariates. Furthermore, lifestyle habit variables were incorporated into the models based on the characteristics of each sampling location (e.g., frequency of airing and changing bed linens for beds, desk cleaning frequency for desks, floor sweeping frequency for floors). In the GLM analyses, to address the high prevalence of zero values (zero-inflation) in the relative abundance data, zero values were replaced with half of the minimum non-zero values that were observed for each respective species. This widely accepted pseudo-count addition method is essential for compositional microbiome data to avoid undefined logarithmic transformations in the subsequent log-link Gamma distribution models, while effectively minimizing distortion to the dataset’s underlying distribution. A Gamma distribution with a log-link function was selected for model fitting. Odds ratio (ORs) and regression coefficients (β), accompanied by their 95% confidence intervals are recorded to show the target associations in the logistic regression analyses and GLM analyses, respectively. A statistical significance threshold of *p* < 0.05 was applied for all analyses. The statistical analyses were performed using SPSS Statistics (version 27.0). Figures were generated using Origin software (version 2024).

## 3. Results

### 3.1. Basic Status of Indoor Ventilation

Table 1 shows the cumulative nighttime AERs from 1 to 7 days before sampling and the overall ventilation compliance rates, which ranged from 43.2% to 52.1%. The ventilation conditions had significant seasonal differences, with spring showing the highest compliance rates (58.3–69.4%), while the rates in summer, autumn, and winter were mostly below 50%. Additionally, the median AER in spring consistently remained above the compliance threshold, whereas the medians in the other three seasons predominantly fell below or near this benchmark (Figure S1).

### 3.2. Pathogenic Bacteria and Fungi Communities

Table 2 details the six pathogenic bacterial genera and 13 pathogenic fungal genera across the sampling sites, cross-referenced with the WHO priority lists.

For the pathogenic bacterial communities, *Serratia* and *Enterobacter* were the genera with the highest detection rates. The detection rates of both *Serratia* and *Enterobacter* were significantly higher in floor dust samples compared to bed and desk surface dust samples. *Salmonella* exhibited its highest detection frequency (21.4%) in desk samples. The detection rates of the remaining bacterial genera were below 5%.

Regarding the pathogenic fungal communities, *Fusarium* and *Acremonium* were detected in nearly all samples, exhibiting extremely high detection rates (92.3–99.5%). Four species in the *Candida* genus were detected, and *Candida parapsilosis* and *Candida tropicalis* generally showed high detection rates, particularly in bed and floor samples. Notably, the detection rate of *Candida parapsilosis* was substantially higher on bed surface dusts (68.8%) and floor dusts (71.7%) than on desk surface dusts (49.7%). The detection rates of the remaining fungal genera/species, such as *Scedosporium* and *Pichia kudriavzevii*, were below 13% (Table 2).

### 3.3. Associations Between AER with Pathogenic Bacteria and Fungi

**Figure 2:**
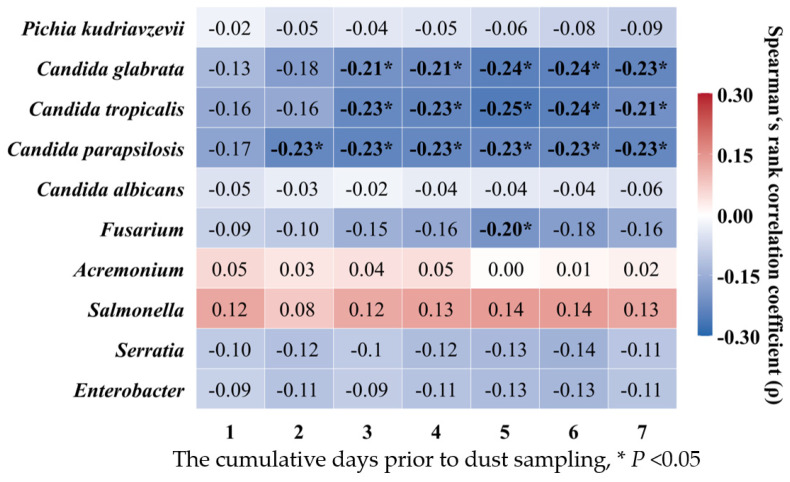
The correlations of relative abundances of pathogenic bacterial genera, fungal genera, and fungal species in the bed dust with nighttime air exchange rates (AERs) in the cumulative 1–7 days prior to dust sampling.

Cumulative AERs (1–7 days prior to sampling) generally exhibited negative correlations with the relative abundances of most pathogens (Figure 2). However, these correlations only reached statistical significance among pathogens from bed dust (Figure 2), and no significant correlation was found among pathogens in floor and desk dust (Figure S2). In bed dust samples, the relative abundances of *Candida glabrata*, *Candida tropicalis*, and *Candida parapsilosis* showed significant negative correlations with cumulative AERs over multiple periods before sampling (particularly 3 to 7 days), with correlation coefficients (ρ) ranging from −0.21 to −0.25. *Fusarium* in bed dust also exhibited a significant negative correlation (ρ = −0.20) with the cumulative AERs in five days before dust sampling.

**Figure 3:**
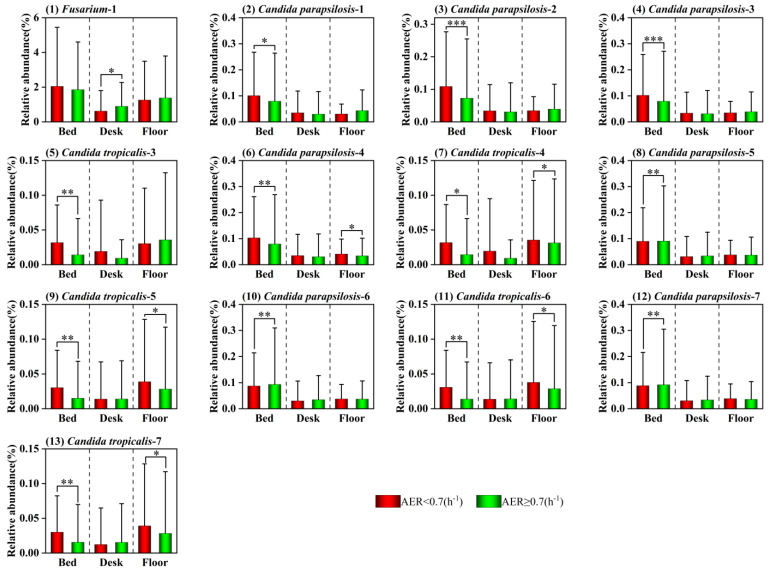
Differential analysis of relative abundances of representative key pathogenic bacteria and fungi at different sampling sites (bed, desk, floor) between air exchange rate (AER) groups (<0.7 h^−1^ vs. ≥0.7 h^−1^). The number suffix after each microbial taxon (e.g., “−1” to “−7”) indicates the day prior to sampling for which the AER was assessed. * *p* < 0.05, ** *p* < 0.01, *** *p* < 0.001.

Figure 3 and Figure S3 show the differences in relative abundances of pathogens in the three sampling sites between non-compliant ventilated dormitories (AER < 0.7 h^−1^) and compliant ventilated dormitories (AER ≥ 0.7 h^−1^). Among the 10 target pathogens analyzed, statistically significant differences in relative abundances were observed only for specific pathogens, at specific locations, and for specific days before the dust sampling. For pathogenic bacteria, the relative abundance of *Serratia* was generally higher across beds, desks, and floors in the dormitories without compliant ventilation on 2–5 days prior to the dust sampling. The differences reached statistical significances among pathogenic bacteria from desk (2–4 days) and floor (4–5 days) dust samples. Conversely, the relative abundance of *Salmonella* in bed dust was significantly higher in the dormitories with compliant ventilation on six days before the dust sampling. Regarding pathogenic fungi, *Acremonium* and *Fusarium* in the desk dust exhibited a pattern of significantly higher relative abundance in the dormitories with compliant ventilation across multiple time periods (e.g., *Acremonium* for the first day and 3–7 days; *Fusarium* for the first day prior to dust sampling). In contrast, several *Candida* from bed surface dust, particularly *Candida parapsilosis* and *Candida tropicalis*, showed significantly lower relative abundance in the dormitories with compliant ventilation across multiple periods (e.g., *Candida parapsilosis* for 1–4 days; *Candida tropicalis* for 3–7 days). A similar relationship was observed for *Candida tropicalis* in floor dust samples (4–7 days). However, the relative abundance of *Pichia kudriavzevii* in floor dust samples was significantly lower in the dormitories with compliant ventilation from 5 to 7 days before sampling.

In the logistic regression analyses, five pathogens exhibited statistically significant associations with cumulative AER at specific locations (Table 3, Supplemental Table S2). The association of cumulative ventilation with the pathogen detection rate varied significantly across species and sampling locations. Among the pathogenic bacteria, the detection rate of *Serratia* in bed dust significantly decreased with increasing cumulative AER over the 5 days preceding sampling. Conversely, the detection rate of *Salmonella* in bed dust significantly increased with higher AERs. For the pathogenic fungi, the detection rate of *Candida parapsilosis* in bed dust showed a significantly negative associations with cumulative AER across all 1–7 days before dust sampling, whereas its detection possibility on desks exhibited a positive association with cumulative AERs. Meanwhile, *Candida tropicalis* showed negative associations in both bed and floor dusts. The detection rate of *Fusarium* in bed dust was negatively associated with the AER in the first day before dust sampling.

In the multivariable generalized linear regression analyses (Table 4, Supplemental Table S3), the association of cumulative AER on the relative abundances of pathogens also exhibited significant species and spatial specificity, and temporal gradient effects were demonstrated for some pathogens.

Among the pathogenic bacteria, the relative abundance of *Salmonella* in bed dust showed a significant positive association with cumulative AERs. The regression coefficient (β) progressively increased with longer time windows (from 0.27 for the first day to 0.33 for 7 days). Conversely, the relative abundance of *Enterobacter* in desk dust was significantly and negatively associated with the cumulative AER, and this negative association strengthened as the time window extended.

The temporal cumulative effect was more prevalent among the pathogenic fungi (Table 4, Supplemental Table S3). The relative abundance of *Candida tropicalis* on bed dust showed a significant negative association with cumulative AER on 3–7 days prior to the dust sampling. Similarly, the strength of the negative association (absolute *β* value) for *Candida glabrata* in bed dust increased significantly from 1 to 3 days. *Fusarium* abundance in bed and desk dust also displayed a similar pattern, and its negative associations with cumulative AER emerged from 4 days and intensified over time.

## 4. Discussion

This study systematically evaluated the associations of indoor cumulative ventilation with WHO priority pathogens in settled dust of university dormitories. The principal findings indicate that the modulatory associations of the indoor nighttime ventilation with both the relative abundances and detection rates of pathogens are not merely inhibitory or promotive but rather exhibits a complex pattern highly dependent on the species type, sampling location, and cumulative ventilation days.

Our study revealed a distinct spatial specificity in the distribution of WHO priority pathogens in the dormitory floor, desk, and bed settled dusts, a pattern likely shaped by the combined influence of surface functions and human activities. For instance, *Serratia* and *Enterobacter* exhibited their highest detection rates on floors. This aligns with findings by Liu et al., who reported *Serratia marcescens* as a dominant pathogen on residential kitchen surfaces [54], suggesting that high-frequency contact surfaces are prone to microbial contamination. For the fungal communities, the widespread presences of *Fusarium* and *Acremonium* are consistent with studies detecting these genera in air conditioning filters [55] and in dormitory wall surface samples [56]. However, Fan et al. reported detection rates exceeding 50% for *Cladosporium*, *Aspergillus*, and *Penicillium* in residential dust, while *Fusarium* was not detected [57]. This notable discrepancy in fungal composition compared to our findings may be attributed to inherent differences between residential and dormitory environments. The high detection rates of Candida species, such as *Candida parapsilosis*, in bed dust further underscore the role of direct human contact in shaping this intimate micro-environment. Compared to clinical or older residential settings [58,59,60,61,62], our newly constructed dormitories lacked widespread detection of critical pathogens (e.g., *Mycobacterium tuberculosis* or *Aspergillus fumigatus*). As a possible explanation, this distinctive, lower-risk microbial profile likely stems from the healthier, homogeneous student population and reduced colonization risks associated with the newer building.

In evaluating the associations of cumulative ventilation before the sampling with pathogen presences, the species and spatial specificity associations suggest that indoor sustained ventilation may modulate the settlement and retention of human-associated microbiota on bed (near-body) surfaces [63]. Recent evidence aligns with this observation: natural ventilation actively reduces indoor pathogen prevalence [48], whereas elevated CO_2_ correlates positively with airborne rhinovirus bioaerosols [64]. These findings collectively reinforce the crucial role of ventilation in modulating microbial exposure.

Our results indicate that generally stronger and more stable associations were observed in bed dust compared to floors or desks. We attribute this discrepancy to the distinct functional and physical layouts of these surfaces: the bed environment is relatively static and experiences prolonged human occupancy during sleeping hours. In contrast, the microbial community on desks and floors is likely dominated by frequent, transient activities and mechanical disturbances that may mask the baseline effects of ventilation. This masking effect of disturbances also explains why short-term window opening often fails to significantly alter the overall microbial community [19].

Our results demonstrated that the cumulative AER exhibited contrasting association patterns with different pathogens. Specifically, the relative abundances of *Candida tropicalis*, *Candida glabrata*, and *Enterobacter* showed stable negative associations with longer-term cumulative AERs. This underscores that single-day or short-duration ventilation is often insufficient, whereas sustained cumulative ventilation provides substantial mitigation, aligning with previous findings [22,44]. Since *Candida* spp. are closely associated with human shedding [65], continuous fresh air exchange can persistently lower the local indoor temperature and relative humidity, thereby disrupting the micro-climatic conditions necessary for their survival and proliferation [66,67]. Similarly, the inhibition of *Enterobacter* is consistent with the established role of ventilation in restricting specific bacterial growth [68]. In addition, our data revealed that the relative abundance of *Salmonella* on beds and desks, as well as *Candida albicans* on floors, increased following higher cumulative AERs. First, although speculative, while introducing fresh air, increased ventilation may concurrently facilitate the continuous ingress of outdoor-origin microorganisms into the dormitory. Other field measurements indirectly support this hypothesis, showing that enhanced natural ventilation elevates both outdoor-origin bacteria [69] and indoor fungal diversity [70]. Second, instead of direct outdoor introduction, enhanced ventilation might drive the mechanical resuspension of previously settled dust [71], subsequently re-depositing it onto high-exposure surfaces like beds and desks.

Although this study systematically revealed the cumulative inhibitory associations and spatial specificity of ventilation with some pathogens, the observed statistically significant correlation coefficients between AER and specific pathogens (e.g., ρ ranging from −0.25 to 0.20 in Figure 2 and Figure S2) are crucially relatively weak. Ventilation levels assessed based on single-day or shorter time windows were not a primary driver of their distribution. This finding aligns with the conclusion drawn by Liu et al. from residential dust, where ventilation showed weak associations with pathogens [62]. Furthermore, the relative abundance of pathogens in settled dust is simultaneously driven by stochastic occupant shedding, surface cleaning frequency, and background microclimate [32,66,72,73]. The emergence of these weak but stable correlations underscores that cumulative ventilation exerts a consistent modulatory baseline association amidst a highly noisy environmental background. Consequently, viewing ventilation as a universal microbial control measure is unrealistic. It must be integrated with comprehensive strategies such as source control and regular cleaning, consistent with the assertion by Gilkeson et al. [40].

Several limitations should be acknowledged in this study. First, the estimation of ventilation rates relied on the CO_2_ concentration step-up method to derive air exchange rates (AERs) during nighttime. Specifically, settled dust accumulates continuously over 24 h, whereas our AER data strictly reflects nighttime ventilation conditions. Second, direct measurement of airflow velocity or ventilation pathways was not conducted, which may introduce some degree of error. Third, the reliance on relative abundance data from sequencing is a critical limitation; observed microbial shifts reflect proportional changes rather than absolute quantitative variations in pathogen biomass. Fourth, the absence of concurrent outdoor microbial air sampling restricts our ability to definitively trace pathogen sources. Lastly, although potential confounders such as indoor air temperature, relative humidity, and lifestyle habits were controlled, the inherent complexity of microbial communities might still influence a fully comprehensive interpretation. Furthermore, while this study observed opposing associations of ventilation with different pathogens, it could not precisely trace their specific sources (e.g., whether originating from indoor or outdoor environments). This limitation restricts a more mechanistic interpretation of the bidirectional associations of ventilation. Concurrently, the study focused solely on a subset of species from the WHO priority pathogen lists and did not encompass all potential pathogenic microorganisms. Future research could integrate more precise ventilation monitoring techniques, simultaneous indoor and outdoor microbial sampling, metagenomic functional analysis, and longitudinal study designs to more comprehensively elucidate the sources and regulatory mechanisms of ventilation on indoor microbial pollution risks.

## 5. Conclusions

This study elucidates the associations of cumulative indoor natural ventilation with the distribution of WHO priority pathogens in settled dust from dormitory beds, desks, and floors. We found that increased air exchange rates (AERs) correlated with lower relative abundance and detection possibility of specific Candida species (e.g., *Candida tropicalis* and *Candida glabrata*) on bed and floor surfaces. Conversely, the relative abundances of certain pathogens, such as Salmonella, correlated positively with higher AERs. Because these contrasting patterns reflect complex environmental associations rather than direct causation, ventilation should not be viewed as a universal pathogen-control strategy. Instead, its modulatory role is highly species-specific and surface-specific. Therefore, precision dormitory management requires integrating sustained daily ventilation with regular surface cleaning and near-body source control (e.g., frequent laundering of bed linens). Finally, future studies should incorporate concurrent outdoor microbial sampling and precise source tracking to comprehensively elucidate the origins and transmission pathways of indoor pathogens.

## Figures and Tables

**Table 1:** The nighttime air exchange rates (AERs) and compliance rates in different periods.

Period	Mean ± SD, (h^−1^)	Compliance (AER ≥ 0.7 h^−1^) Rate, %				
		Spring	Summer	Autumn	Winter	All
1 day before sampling day	1.28 ± 1.64	58.3	41.7	66.7	41.7	52.1
2 days before sampling day	1.23 ± 1.58	66.7	41.7	37.5	33.3	44.8
3 days before sampling day	1.27 ± 1.59	69.4	38.9	36.1	30.6	43.8
4 days before sampling day	1.22 ± 1.57	66.7	37.5	37.5	31.3	43.2
5 days before sampling day	1.26 ± 1.61	65.0	36.7	38.3	38.3	44.6
6 days before sampling day	1.26 ± 1.56	65.3	36.1	40.3	40.3	45.5
7 days before sampling day	1.27 ± 1.54	65.5	38.1	40.5	42.9	46.7

SD, standard deviation.

**Table 2:** Detection rates of pathogenic bacterial genera, fungal genera, and fungal species.

	Grade	Detection Rate, %		
		Bed	Desk	Floor
Bacteria (genus)				
***Serratia* **	**1**	**19.7**	**27.8**	**52.9**
***Enterobacter* **	**1**	**8.4**	**19.3**	**23.0**
***Salmonella* **	**2**	**6.7**	**21.4**	**13.9**
*Morganella*	1	1.1	1.1	2.7
*Proteus*	1	1.7	3.7	4.8
*Citrobacter*	1	0.0	0.0	0.5
Fungi (genus)				
***Fusarium* **	**2**	**97.7**	**94.5**	**99.5**
***Acremonium* **	**2**	**93.8**	**92.3**	**96.3**
*Choanephora*	2	0.6	0.0	0.0
*Rhizopus*	2	0.0	0.5	0.0
*Lichtheimia*	2	0.0	0.5	0.0
*Madurella*	2	0.0	0.0	0.5
*Scedosporium*	3	0.6	1.1	1.1
Fungi (species)				
***Candida albicans* **	**1**	**8.5**	**2.2**	**6.4**
***Candida parapsilosis* **	**2**	**68.8**	**49.7**	**71.7**
***Candida tropicalis* **	**2**	**44.9**	**23.5**	**55.1**
***Candida glabrata* **	**2**	**6.3**	**1.6**	**4.3**
***Pichia kudriavzevii* **	**3**	**6.8**	**12.6**	**8.0**
*Talaromyces marneffei*	3	0.6	0.0	0.0

Note: Grade indicates the hazard classification levels for respective bacterial genera and fungal taxa (genera/species) in the WHO priority pathogen list; the bold pathogens were included in the following association analyses.

**Table 3:** The significant associations between air exchange rates (AERs) during the cumulative 1–7 days before dust sampling and pathogenic bacteria and fungi.

		Odds Ratio (Detected vs. Undetected)						
		1 Day	2 Days	3 Days	4 Days	5 Days	6 Days	7 Days
*Serratia*	Bed					0.65 *		
*Salmonella*	Bed	1.42 *	1.60 *	1.81 *	1.73 *			
	Desk		1.61 **	1.65 **	1.64 **	1.47 *	1.48 *	1.48 *
	Floor	1.29 *						
*Fusarium*	Bed	0.56 *						
*Candida parapsilosis*	Bed	0.80 *	0.76 *	0.67 **	0.69 *	0.70 *	0.69 *	0.70 *
	Desk			1.44 *	1.47 *	1.41 *	1.47 *	1.44 *
*Candida tropicalis*	Bed			0.69 *	0.67 *	0.64 **	0.65 **	0.64 **
	Floor		0.73 *	0.66 **	0.64 **	0.59 **	0.60 **	0.61 **

Note: * *p* < 0.05, ** *p* < 0.01; models were adjusted for indoor air temperature, relative humidity, and lifestyle habits.

**Table 4:** The significant associations between air exchange rates and relative abundances of pathogenic bacteria and fungi in the multivariable generalized linear regression analyses.

		Regression Coefficient, *β*						
		1 Day	2 Days	3 Days	4 Days	5 Days	6 Days	7 Days
*Enterobacter*	bed			−0.33 *	−0.35 **	−0.33 **	−0.34 **	−0.30 *
	desk				−0.38 *	−0.41 *	−0.49 **	−0.55 **
*Serratia*	desk		−0.54 *	−0.89 ***	−0.60 *	−0.61 *		
	floor		−0.36 *					
*Salmonella*	bed	0.27 **	0.30 *	0.34 *	0.34 *	0.32 *	0.33 *	0.33 *
*Acremonium*	bed	0.05 *						
*Fusarium*	bed				−0.18 *	−0.23 **	−0.26 **	−0.29 **
	desk				−0.20 *	−0.20 *	−0.23 *	−0.24 *
*Candida albicans*	bed		1.23 ***					
	desk		0.28 *					
	floor			0.16 *		0.15 *	0.14 *	0.17 *
*Candida tropicalis*	bed			−0.42 **	−0.46 **	−0.48 **	−0.49 **	−0.50 **
	floor				−0.54 **	−0.68 ***	−0.69 ***	−0.70 ***
*Candida glabrata*	bed	−0.31 ***	−0.49 ***	−0.64 ***	−0.54 ***	−0.52 ***	−0.52 ***	−0.53 ***
	floor		−0.28 *	−0.37 **	−0.36 *	−0.33 *	−0.32 *	−0.32 *
*Pichia kudriavzevii*	floor							−0.25 *

Note: * *p* < 0.05, ** *p* < 0.01, *** *p* < 0.001; models were adjusted for indoor air temperature, relative humidity, and lifestyle habits.

## Data Availability

The original data of 16s rRNA gene sequencing have been deposited in NCBI under accession number PRJNA1393291 and PRJNA1393299.

## Supplementary Materials

The following supporting information can be downloaded at https://www.mdpi.com/article/10.3390/microorganisms14071521/s1. Figure S1: The nighttime air exchange rate (AER) during 1–7 days before sampling in different seasons; Figure S2: The correlations of relative abundances of pathogenic bacterial genera, fungal genera, and fungal species in the floor and desk dusts with nighttime air exchange rates (AERs) in the cumulative 1–7 days prior to dust sampling; Figure S3: Differential analysis of relative abundances of additional pathogenic bacteria and fungi at different sampling sites (bed, desk, floor) between air exchange rate (AER) groups (<0.7 h^−1^ vs. ≥0.7 h^−1^). The number suffix after each microbial taxon (e.g., “_1” to “_7”) indicates the day prior to sampling for which the AER was assessed. * *p* < 0.05, ** *p* < 0.01, *** *p* < 0.001; Table S1: Characteristics of occupants’ habits in the studied dormitories; Table S2: The associations between air exchange rates (AERs) during the cumulative 1–7 days before dust sampling and pathogenic bacteria and fungi in the multivariate logistic regression analyses; Table S3: The significant associations between air exchange rate and relative abundances of pathogenic bacteria and fungi in the multivariable generalized linear regression analyses.
