# Supplementary material for: Associations of Indoor Nighttime Ventilation with the Relative Abundances of Typical Pathogenic Bacteria and Fungi in Settled Dusts from Floor, Desk, and Bed of University Dormitories
Source: Microorganisms. 2026 Jul 12;14(7):1521. doi: 10.3390/microorganisms14071521 (PMC13413791; doi:10.3390/microorganisms14071521)
Supplement: Supplementary file 1 [file microorganisms-14-01521-s001.zip › microorganisms-4359801-supplementary.pdf]

Supplemental Materials

Associations of Indoor Nighttime Ventilation with the Relative Abundances of Typical Pathogenic Bacteria and Fungi in Settled Dusts from Floor, Desk, and Bed of University Dormitories

Wei Liu[1, 2], Wangjin Lai[1, 2], Yu Zhang[1, 2], Jinze Du[1, 2], Ying Chen[1, 2], Zhi Li[1, 2], Nan Zhang[3, 4], Jiao Cai[1, 2, *]

1. School of Civil and Hydraulic Engineering, Chongqing University of Science and Technology, Chongqing, 401331, China

2. Institute for Health and Environment, Chongqing University of Science and Technology, Chongqing, 401331, China

3. Beijing Key Laboratory of Green Built Environment and Energy Efficient Technology, Beijing University of Technology, Beijing, 100124, China

4. Chongqing Research Institute of Beijing University of Technology, Chongqing, 401100, China

* **Corresponding author**. E-mail address: caijiao@cqust.edu.cn

Section S1. Example Calculation of the Air Exchange Rate (AER)

The calculation of the nighttime AER is based on the CO_2 mass balance equation, employing a non-linear curve-fitting method to ensure robustness against sensor noise. The detailed calculation procedure for a typical dormitory is presented below.

Step 1: Determination of the CO_2 Emission Rate, $F(t)$

The continuous CO_2 emission rate (F_{CO2}) for each occupant during sleep was estimated using the following empirical biometric equation:

$$F_{CO2} = \frac{0.201 \times RQ \times M \times H^{0.725} \times W^{0.425}}{21 \times (0.23 \times RQ + 0.77)}$$

Where:

RQ : Respiratory quotient (dimensionless), taken as 0.83 for resting/sleeping conditions.

M : Metabolic rate ($\mathrm{W/m^2}$), taken as $40\ \mathrm{W/m^2}$ during sleep.

H : Height of the occupant (m).

W : Weight of the occupant (kg).

Taking a specific 4-person dormitory as an example (Room Volume V = 64 m^3), the biometric data of the four occupants were:Occupant 1 (1.56 m, 56 kg), Occupant 2 (1.63 m, 55 kg), Occupant 3 (1.57 m, 47 kg), and Occupant 4 (1.68 m, 59 kg).Substituting these values into the equation yields individual emission rates of 0.0025, 0.0026, 0.0024, and 0.0027 L/s, respectively. The total CO_2 emission rate for the dormitory, $F(t)$ is the sum of these values, equaling 0.0102 L/s (equivalent to 0.0367 $\mathrm{m^3/h}$).

Step 2: The Integral Mass Balance Equation

Assuming the indoor air is well-mixed, the dynamic change in indoor CO_2 concentration is governed by the mass balance:

$$V\frac{dC_t}{dt} = F(t) + AER \cdot V \cdot (C_{out} - C_t)$$

By integrating this differential equation over the nighttime build-up period (from initial time $t = 0$ to time t), the theoretical indoor CO_2 concentration at any given time, C_t (ppm), can be expressed as:

$$C_t = C_{out} + \frac{F(t)}{AER \cdot V} - \left(\frac{F(t)}{AER \cdot V} - (C_0 - C_{out})\right)e^{-AER \cdot t}$$

Where:

C_0 : Initial indoor CO_2 concentration at the start of the build-up phase (ppm).

C_{out} : Outdoor CO_2 background concentration (assumed constant at approximately 40 ppm).

V : Room volume (m^3).

AER : Air exchange rate (h^{-1}).

t : Elapsed time (h).

Step 3: Curve-Fitting to Solve for AER

Instead of solving algebraically at a single time point, the theoretical concentration C_t was calculated for

every 10-minute interval throughout the sleep period (22:00 to 8:00) using a presumed AER value. The Generalized Reduced Gradient (GRG) Nonlinear solver function in Microsoft Excel was then utilized to iteratively adjust the AER to minimize the Sum of Squared Errors (SSE) between the theoretically predicted concentrations ($C_{predicted}$) and the actual continuous sensor readings ($C_{measured}$):

$$\text{Minimize SSE} = \sum_{t=0}^{n} (C_{measured,t} - C_{predicted,t})^2$$

The AER value that generated the best-fitting exponential curve corresponding to the minimum SSE was adopted as the final effective nighttime air exchange rate for that specific dormitory.

Table S1. Characteristics of occupants' habits in the studied dormitories.

Home characteristics		Frequency (%) N = 187
Bedding sun-drying frequency	Seldom	41.7% (78)
	Several times a month	55.1% (103)
	Several times a week	3.3% (6)
Bedding replacement frequency	Less than once a month	26.7% (50)
	Once a month	52.9% (99)
	Once every two weeks	20.3% (38)
Daily bed-making	Yes	29.9% (56)
	No	70.1% (131)
Bed curtain usage	Yes	65.8% (123)
	No	34.2% (64)
Desk cleaning frequency	Once a month	53.5% (100)
	Once every two weeks	26.2% (49)
	Once a week	13.4% (25)
	2–3 times a week	7.0% (13)
Sweeping frequency	Once a month	22.5% (42)
	Once every two weeks	22.5% (42)
	Once a week	24.6% (46)
	2–3 times a week	30.5% (57)
Mopping frequency	Once a month	51.3% (96)
	Once every two weeks	20.3% (38)
	Once a week	16.0% (30)
	2–3 times a week	12.3% (23)
Daily occupancy duration	< 10h	42.2% (79)
	10 - 12h	27.8% (52)
	> 12h	29.9% (56)
Indoor plants	Yes	72.2% (135)
	No	27.8% (52)
Indoor smoking	Yes	27.3% (51)
	No	72.7% (136)

Table S2. The associations between air exchange rates (AERs) during the cumulative 1-7 days before dust sampling and pathogenic bacteria and fungi in the multivariate logistic regression analyses.

		Odds ratio (detected *vs.* un-detected)						
		1 day	2 days	3 days	4 days	5 days	6 days	7 days
Enterobacter	bed	0.73 (0.40,1.07)	0.63 (0.32,1.05)	0.67 (0.36,1.10)	0.63 (0.30,1.08)	0.62 (0.28,1.10)	0.59 (0.25,1.09)	0.68 (0.32,1.20)
	desk	0.97 (0.77,1.19)	1.00 (0.74,1.31)	0.83 (0.58,1.15)	0.74 (0.49,1.05)	0.74 (0.50,1.06)	0.74 (0.49,1.07)	0.75 (0.49,1.09)
	floor	0.75 (0.52,0.98)	0.78 (0.55,1.05)	0.80 (0.57,1.10)	0.85 (0.60,1.16)	0.83 (0.59,1.14)	0.94 (0.67,1.28)	0.96 (0.68,1.32)
Serratia	bed	0.90 (0.65,1.14)	0.74 (0.49,1.04)	0.73 (0.48,1.04)	0.69 (0.44,0.99)	**0.65 (0.41,0.95)** *	0.64 (0.40,0.95)	0.70 (0.45,1.02)
	desk	0.86 (0.66,1.07)	0.73 (0.51,0.98)	0.74 (0.53,1.01)	0.79 (0.56,1.08)	0.78 (0.55,1.06)	0.84 (0.60,1.16)	0.87 (0.62,1.19)
	floor	0.98 (0.79,1.18)	0.94 (0.73,1.20)	0.87 (0.66,1.14)	0.82 (0.62,1.08)	0.80 (0.61,1.05)	0.82 (0.62,1.07)	0.82 (0.62,1.07)
Salmonella	bed	**1.42 (1.08,1.92)** *	**1.60 (1.04,2.51)** *	**1.81 (1.06,3.38)** *	**1.73 (1.02,3.03)** *	1.53 (0.94,2.50)	1.54 (0.92,2.54)	1.50 (0.90,2.45)
	desk	1.22 (0.99,1.52)	**1.61 (1.20,2.21)** **	**1.65 (1.15,2.42)** **	**1.64 (1.16,2.35)** **	**1.47 (1.06,2.04)** *	**1.48 (1.06,2.09)** *	**1.48 (1.06,2.07)** *
	floor	**1.29 (1.01,1.64)** *	1.23 (0.87,1.69)	1.39 (0.93,2.08)	1.35 (0.91,1.98)	1.23 (0.85,1.77)	1.21 (0.82,1.75)	1.23 (0.84,1.76)
Acremonium	bed	0.85 (0.61,1.34)	0.98 (0.58,1.97)	1.37 (0.73,3.09)	1.50 (0.76,3.94)	1.39 (0.75,3.34)	1.36 (0.72,3.26)	1.34 (0.71,3.23)
	desk	1.18 (0.80,2.40)	1.69 (0.92,4.19)	2.07 (1.05,5.49)	2.29 (1.07,7.05)	1.83 (0.94,4.65)	1.98 (0.97,5.11)	2.07 (0.98,5.55)
	floor	0.74 (0.52,1.06)	0.63 (0.35,1.09)	0.68 (0.32,1.35)	0.80 (0.42,1.56)	0.84 (0.46,1.64)	0.90 (0.49,1.80)	0.94 (0.52,1.89)
Fusarium	bed	**0.56 (0.28,0.93)** *	0.44 (0.16,0.98)	0.52 (0.14,1.44)	0.62 (0.22,1.77)	0.67 (0.28,1.77)	0.68 (0.28,1.86)	0.74 (0.32,2.06)
	desk	6.02 (1.32,71.25)	2.05 (0.89,8.35)	2.31 (0.95,9.61)	2.53 (0.96,12.90)	2.15 (0.90,8.32)	2.38 (0.95,9.51)	2.52 (0.97,10.44)
	floor	1.11 (0.43,6.87)	0.73 (0.08,3.15)	0.00 (0.00, Inf)	0.44 (0.02,2.11)	0.34 (0.00,1.80)	0.34 (0.00,1.71)	0.36 (0.00,1.61)
Candida albicans	bed	0.76 (0.41,1.09)	0.74 (0.41,1.14)	0.87 (0.52,1.34)	0.89 (0.53,1.38)	0.92 (0.55,1.43)	0.93 (0.54,1.46)	0.93 (0.54,1.44)
	desk	0.28 (0.01,1.10)	1.01 (0.40,1.89)	0.78 (0.25,1.78)	0.70 (0.18,1.68)	0.75 (0.20,1.75)	0.73 (0.19,1.81)	0.80 (0.22,1.95)
	floor	0.70 (0.24,1.26)	1.30 (0.83,2.00)	1.47 (0.83,2.78)	1.34 (0.75,2.38)	1.34 (0.77,2.29)	1.31 (0.73,2.26)	1.36 (0.78,2.33)
Candida parapsilosis	bed	**0.80 (0.64,0.98)** *	**0.76 (0.57,0.98)** *	**0.67 (0.49,0.90)** **	**0.69 (0.51,0.92)** *	**0.70 (0.52,0.92)** *	**0.69 (0.52,0.92)** *	**0.70 (0.52,0.92)** *
	desk	1.17 (0.96,1.44)	1.22 (0.95,1.59)	**1.44 (1.08,1.95)** *	**1.47 (1.09,2.01)** *	**1.41 (1.06,1.92)** *	**1.47 (1.09,2.04)** *	**1.44 (1.07,1.99)** *

	floor	0.99<br>(0.82,1.22)	1.07<br>(0.82,1.40)	1.10<br>(0.83,1.47)	1.12<br>(0.84,1.52)	1.11<br>(0.84,1.52)	1.09<br>(0.81,1.49)	1.11<br>(0.82,1.53)
Candida tropicalis	bed	0.83<br>(0.60,1.05)	0.87<br>(0.65,1.14)	**0.69**<br>**(0.49,0.93)** *	**0.67**<br>**(0.47,0.91)** *	**0.64**<br>**(0.46,0.87)** **	**0.65**<br>**(0.46,0.88)** **	**0.64**<br>**(0.45,0.86)** **
	desk	1.00<br>(0.76,1.24)	0.99<br>(0.72,1.33)	1.01<br>(0.71,1.41)	0.99<br>(0.70,1.39)	0.98<br>(0.69,1.36)	0.97<br>(0.67,1.37)	0.99<br>(0.69,1.38)
	floor	0.87<br>(0.66,1.08)	**0.73**<br>**(0.54,0.96)** *	**0.66**<br>**(0.49,0.88)** **	**0.64**<br>**(0.46,0.85)** **	**0.59**<br>**(0.43,0.79)** **	**0.60**<br>**(0.43,0.81)** **	**0.61**<br>**(0.44,0.82)** **
Candida glabrata	bed	0.45<br>(0.13,0.97)	0.36<br>(0.08,0.91)	0.31<br>(0.05,0.84)	0.27<br>(0.04,0.85)	0.18<br>(0.01,0.76)	0.18<br>(0.01,0.76)	0.18<br>(0.01,0.77)
	desk	0.47<br>(0.01,1.49)	0.40<br>(0.01,1.82)	0.50<br>(0.02,2.23)	0.41<br>(0.01,2.48)	0.32<br>(0.00,2.50)	0.17<br>(0.00,2.33)	0.09<br>(0.00,2.15)
	floor	0.22<br>(0.03,0.78)	0.05<br>(0.00,0.48)	0.03<br>(0.00,0.45)	0.01<br>(0.00,0.30)	0.04<br>(0.00,0.52)	0.09<br>(0.00,0.71)	0.11<br>(0.00,0.74)
Pichia kudriavzevii	bed	0.78<br>(0.36,1.24)	0.70<br>(0.30,1.27)	0.85<br>(0.43,1.49)	0.97<br>(0.51,1.67)	1.02<br>(0.54,1.71)	0.99<br>(0.51,1.70)	0.96<br>(0.49,1.62)
	desk	1.08<br>(0.83,1.35)	1.07<br>(0.75,1.48)	1.12<br>(0.74,1.66)	1.14<br>(0.76,1.68)	1.16<br>(0.78,1.69)	1.22<br>(0.81,1.80)	1.19<br>(0.79,1.75)
	floor	0.55<br>(0.20,1.13)	0.59<br>(0.20,1.24)	0.64<br>(0.25,1.30)	0.61<br>(0.21,1.25)	0.52<br>(0.16,1.12)	0.52<br>(0.16,1.12)	0.49<br>(0.15,1.09)

Note: * $P < 0.05$, ** $P < 0.01$; Models were adjusted for indoor air temperature, relative humidity, and lifestyle habits.

Table S3. The significant associations between air exchange rate and relative abundances of pathogenic bacteria and fungi in the multivariable generalized linear regression analyses.

		Regression coefficient, β						
		1 day	2 days	3 days	4 days	5 days	6 days	7 days
Enterobacter	bed	-0.16	-0.24	**-0.33**	**-0.35**	**-0.33**	**-0.34**	**-0.30**
		(-0.34, 0.08)	(-0.48, 0.05)	**(-0.62, -0.01) ***	**(-0.62, -0.04) ****	**(-0.58, -0.04) ****	**(-0.59, -0.05) ****	**(-0.57, -0.01) ***
	desk	0.00	-0.11	-0.25	**-0.38**	**-0.41**	**-0.49**	**-0.55**
		(-0.28, 0.28)	(-0.35, 0.17)	(-0.54, 0.07)	**(-0.70, -0.03) ***	**(-0.74, -0.05) ***	**(-0.85, -0.10) ****	**(-0.93, -0.11) ****
	floor	-0.11	-0.20	-0.25	-0.24	-0.32	-0.27	-0.24
		(-0.24, 0.28)	(-0.60, 0.20)	(-0.71, 0.21)	(-0.64, 0.22)	(-0.72, 0.13)	(-0.66, 0.18)	(-0.61, 0.19)
Serratia	bed	-0.10	-0.27	-0.27	-0.26	-0.27	-0.28	-0.25
		(-0.30, 0.19)	(-0.55, 0.08)	(-0.65, 0.15)	(-0.62, 0.14)	(-0.60, 0.11)	(-0.60, 0.09)	(-0.56, 0.12)
	desk	-0.23	**-0.54**	**-0.89**	**-0.60**	**-0.61**	-0.55	-0.51
		(-0.67, 0.20)	**(-1.03, -0.04) ***	**(-1.33, -0.44) *****	**(-0.99, -0.16) ***	**(-1.01, -0.13) ***	(-0.96, -0.05)	(-0.93, 0.05)
	floor	-0.38	**-0.36**	-0.51	-0.59	-0.65	-0.55	-0.56
		(-1.18, 0.15)	**(-0.73, 0.00) ***	(-1.27, 0.08)	(-1.47, 0.06)	(-1.61, 0.04)	(-1.40, 0.10)	(-1.44, 0.11)
Salmonella	bed	**0.27**	**0.30**	**0.34**	**0.34**	**0.32**	**0.33**	**0.33**
		**(0.12, 0.46) ****	**(0.10, 0.53) ***	**(0.10, 0.61) ***	**(0.06, 0.65) ***	**(0.02, 0.65) ***	**(0.00, 0.68) ***	**(-0.02, 0.70) ***
	desk	0.10	0.25	0.27	0.27	0.23	0.23	0.28
		(-0.08, 0.34)	(-0.04, 0.60)	(-0.08, 0.64)	(-0.06, 0.63)	(-0.10, 0.60)	(-0.12, 0.62)	(-0.08, 0.69)
	floor	-0.11	0.21	0.19	0.01	-0.20	-0.15	-0.08
		(-0.49, 0.26)	(-0.30, 0.73)	(-0.68, 1.10)	(-0.82, 1.01)	(-0.87, 0.70)	(-0.88, 0.84)	(-0.85, 0.97)
Acremonium	bed	**0.05**	0.08	0.05	0.02	-0.03	-0.03	-0.04
		**(-0.09, 0.22) ***	(-0.10, 0.28)	(-0.15, 0.27)	(-0.18, 0.24)	(-0.22, 0.18)	(-0.23, 0.18)	(-0.23, 0.18)
	desk	0.16	0.12	0.16	0.15	0.12	0.11	0.11
		(0.00, 0.37)	(-0.09, 0.37)	(-0.08, 0.41)	(-0.09, 0.40)	(-0.11, 0.37)	(-0.13, 0.38)	(-0.13, 0.38)
	floor	0.02	0.04	-0.04	-0.09	-0.10	-0.11	-0.10
		(-0.15, 0.23)	(-0.18, 0.30)	(-0.30, 0.23)	(-0.35, 0.20)	(-0.36, 0.18)	(-0.38, 0.18)	(-0.36, 0.18)
Fusarium	bed	-0.06	-0.05	-0.16	**-0.18**	**-0.23**	**-0.26**	**-0.29**
		(-0.16, 0.06)	(-0.22, 0.13)	(-0.35, 0.03)	**(-0.37, 0.02) ***	**(-0.41, -0.04) ****	**(-0.44, -0.06) ****	**(-0.46, -0.09) ****
	desk	0.04	-0.02	-0.13	**-0.20**	**-0.20**	**-0.23**	**-0.24**
		(-0.06, 0.18)	(-0.15, 0.14)	(-0.30, 0.04)	**(-0.36, -0.02) ***	**(-0.37, -0.02) ***	**(-0.41, -0.03) ***	**(-0.43, -0.03) ***
	floor	-0.02	-0.06	-0.08	-0.06	-0.10	-0.12	-0.12
		(-0.16, 0.16)	(-0.24, 0.13)	(-0.27, 0.12)	(-0.24, 0.13)	(-0.26, 0.08)	(-0.29, 0.07)	(-0.29, 0.07)
Candida	bed	-0.22	**1.23**	0.12	0.05	0.13	-0.01	-0.05
albicans		(-0.69, 0.24)	**(0.65, 1.81) *****	(-0.37, 0.62)	(-0.47, 0.58)	(-0.39, 0.65)	(-0.51, 0.50)	(-0.53, 0.43)
	desk	-0.09	**0.28**	0.18	0.12	0.13	0.08	0.07
		(-0.27, 0.16)	**(0.06, 0.53) ***	(-0.15, 0.54)	(-0.25, 0.53)	(-0.22, 0.53)	(-0.28, 0.50)	(-0.29, 0.48)
	floor	-0.04	0.10	**0.16**	0.14	**0.15**	**0.14**	**0.17**
		(-0.14, 0.08)	(-0.04, 0.25)	**(0.02, 0.31) ***	(-0.01, 0.29)	**(0.01, 0.31) ***	**(-0.01, 0.31) ***	**(0.03, 0.32) ***
Candida	bed	-0.04	-0.03	0.01	0.05	0.06	0.06	0.05
parapsilosis		(-0.20, 0.17)	(-0.26, 0.21)	(-0.22, 0.25)	(-0.17, 0.28)	(-0.16, 0.29)	(-0.16, 0.30)	(-0.17, 0.28)
	desk	0.07	0.18	0.25	0.24	0.26	0.24	0.24
		(-0.14, 0.35)	(-0.09, 0.49)	(-0.04, 0.55)	(-0.05, 0.56)	(-0.03, 0.57)	(-0.06, 0.57)	(-0.05, 0.57)

	floor	0.10	0.11	0.06	0.04	-0.02	-0.06	-0.11
		(-0.08, 0.32)	(-0.10, 0.33)	(-0.14, 0.27)	(-0.17, 0.26)	(-0.23, 0.21)	(-0.28, 0.18)	(-0.33, 0.13)
Candida t	bed	-0.16	-0.21	**-0.42**	**-0.46**	**-0.48**	**-0.49**	**-0.50**
ropicalis		(-0.33, 0.05)	(-0.47, 0.09)	**(-0.75, -0.06) ****	**(-0.80, -0.08) ****	**(-0.81, -0.11) ****	**(-0.82, -0.11) ****	**(-0.81, -0.12) ****
	desk	-0.08	0.02	-0.08	-0.05	0.09	0.11	0.14
		(-0.33, 0.29)	(-0.35, 0.49)	(-0.50, 0.40)	(-0.47, 0.43)	(-0.34, 0.61)	(-0.35, 0.69)	(-0.36, 0.77)
	floor	0.13	-0.04	-0.35	**-0.54**	**-0.68**	**-0.69**	**-0.70**
		(-0.14, 0.40)	(-0.42, 0.35)	(-0.74, 0.04)	**(-0.92, -0.10) ****	**(-0.98, -0.32) *****	**(-1.00, -0.33) *****	**(-1.00, -0.34) *****
Candida	bed	**-0.31**	**-0.49**	**-0.64**	**-0.54**	**-0.52**	**-0.52**	**-0.53**
glabrata		**(-0.45, -0.17) *****	**(-0.70, 0.28) *****	**(-0.88, -0.41) *****	**(-0.77, -0.31) *****	**(-0.74, -0.31) *****	**(-0.75, -0.31) *****	**(-0.74, -0.31) *****
	desk	0.00	0.00	-0.01	-0.01	-0.01	-0.01	-0.01
		(-0.02, 0.01)	(-0.02, 0.01)	(-0.02, 0.01)	(-0.02, 0.01)	(-0.02, 0.01)	(-0.02, 0.01)	(-0.02, 0.01)
	floor	-0.18	**-0.28**	**-0.37**	**-0.36**	**-0.33**	**-0.32**	**-0.32**
		(-0.31, 0.02)	**(-0.45, -0.05) ***	**(-0.59, -0.14) ****	**(-0.59, -0.11) ***	**(-0.57, -0.06) ***	**(-0.58, -0.03) ***	**(-0.58, -0.02) ***
Pichia	bed	-0.11	-0.17	-0.17	-0.15	-0.14	-0.15	-0.16
kudriavzevii		(-0.25, 0.07)	(-0.37, 0.07)	(-0.41, 0.10)	(-0.38, 0.11)	(-0.35, 0.10)	(-0.36, 0.09)	(-0.36, 0.07)
	desk	0.10	0.12	0.16	0.16	0.16	0.21	0.22
		(-0.12, 0.42)	(-0.20, 0.53)	(-0.22, 0.57)	(-0.20, 0.57)	(-0.17, 0.56)	(-0.15, 0.63)	(-0.15, 0.65)
	floor	-0.07	-0.11	-0.15	-0.19	-0.22	-0.23	**-0.25**
		(-0.24, 0.14)	(-0.36, 0.18)	(-0.45, 0.16)	(-0.47, 0.12)	(-0.48, 0.06)	(-0.48, 0.05)	**(-0.49, 0.02) ***

Note: * $P < 0.05$, ** $P < 0.01$, *** $P < 0.001$; Models were adjusted for indoor air temperature, relative humidity, and lifestyle habits.

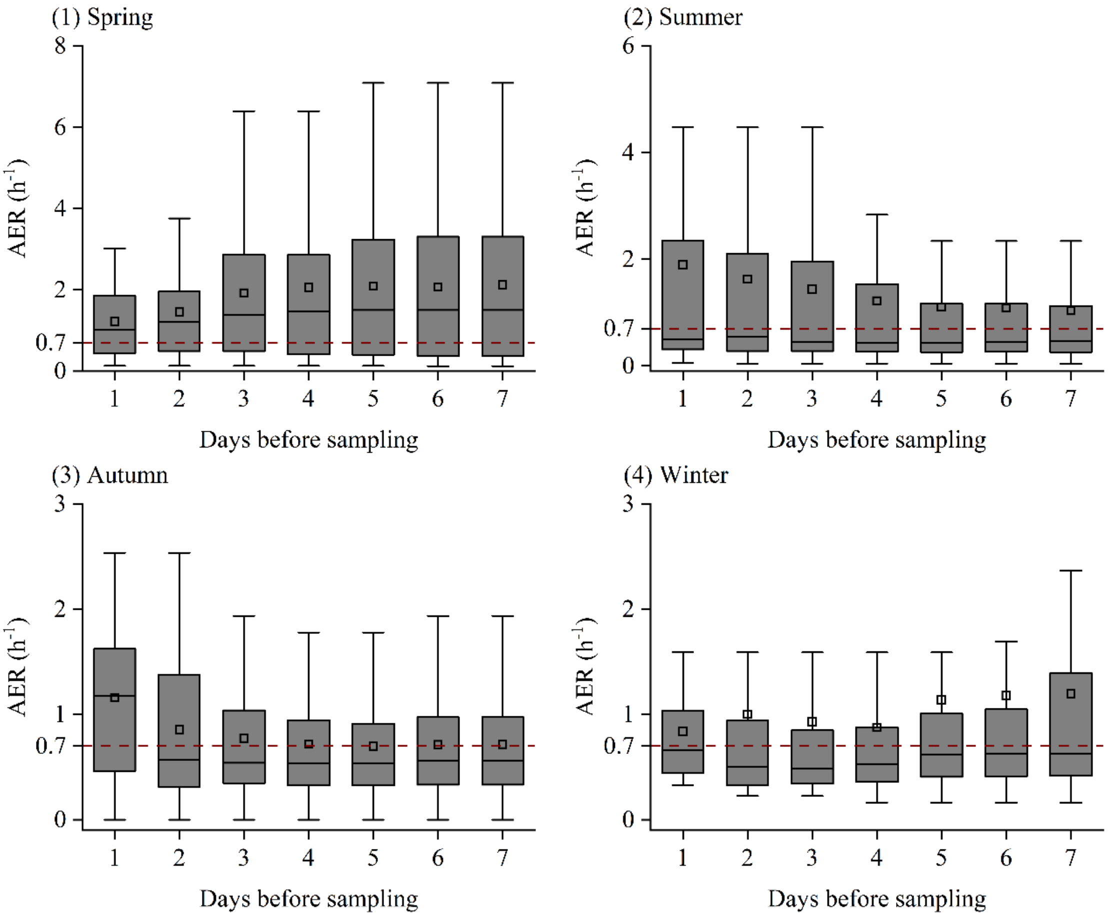

Figure S1. The nighttime air exchange rate (AER) during 1-7 days before sampling in different seasons.

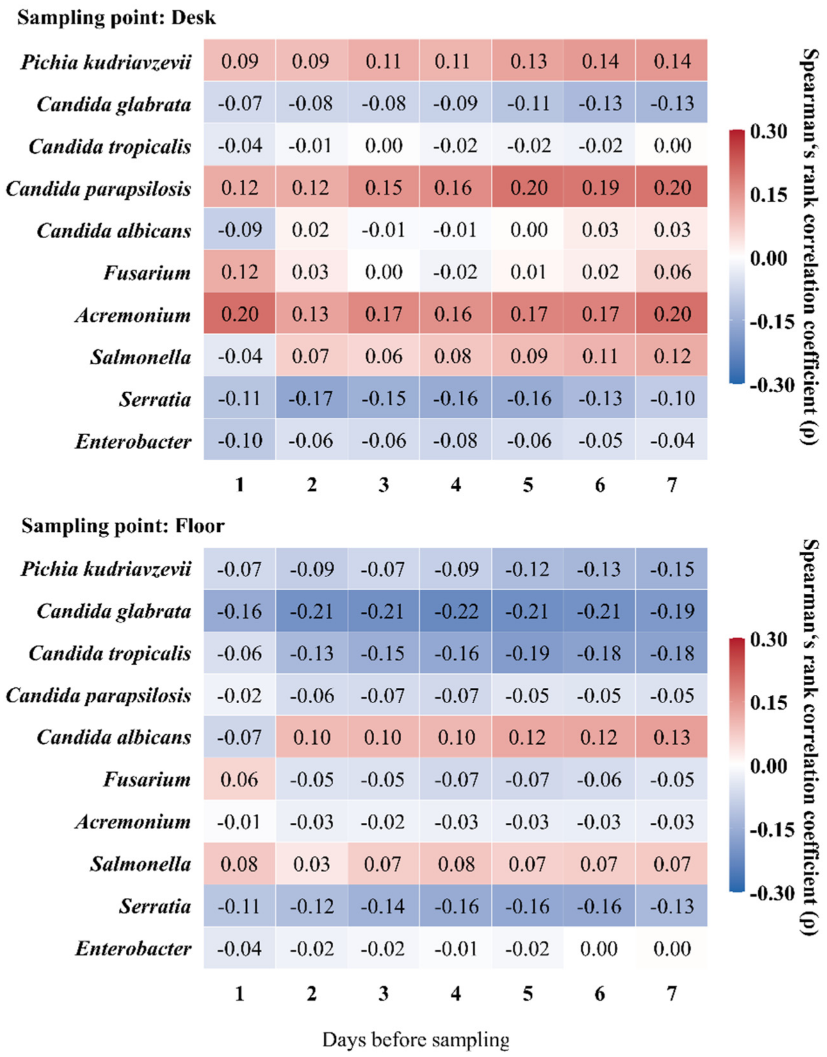

Figure S2. The correlations of relative abundances of pathogenic bacterial genera, fungal genera, and fungal species in the floor and desk dusts with nighttime air exchange rates (AERs) in the cumulative 1-7 days prior to dust sampling.

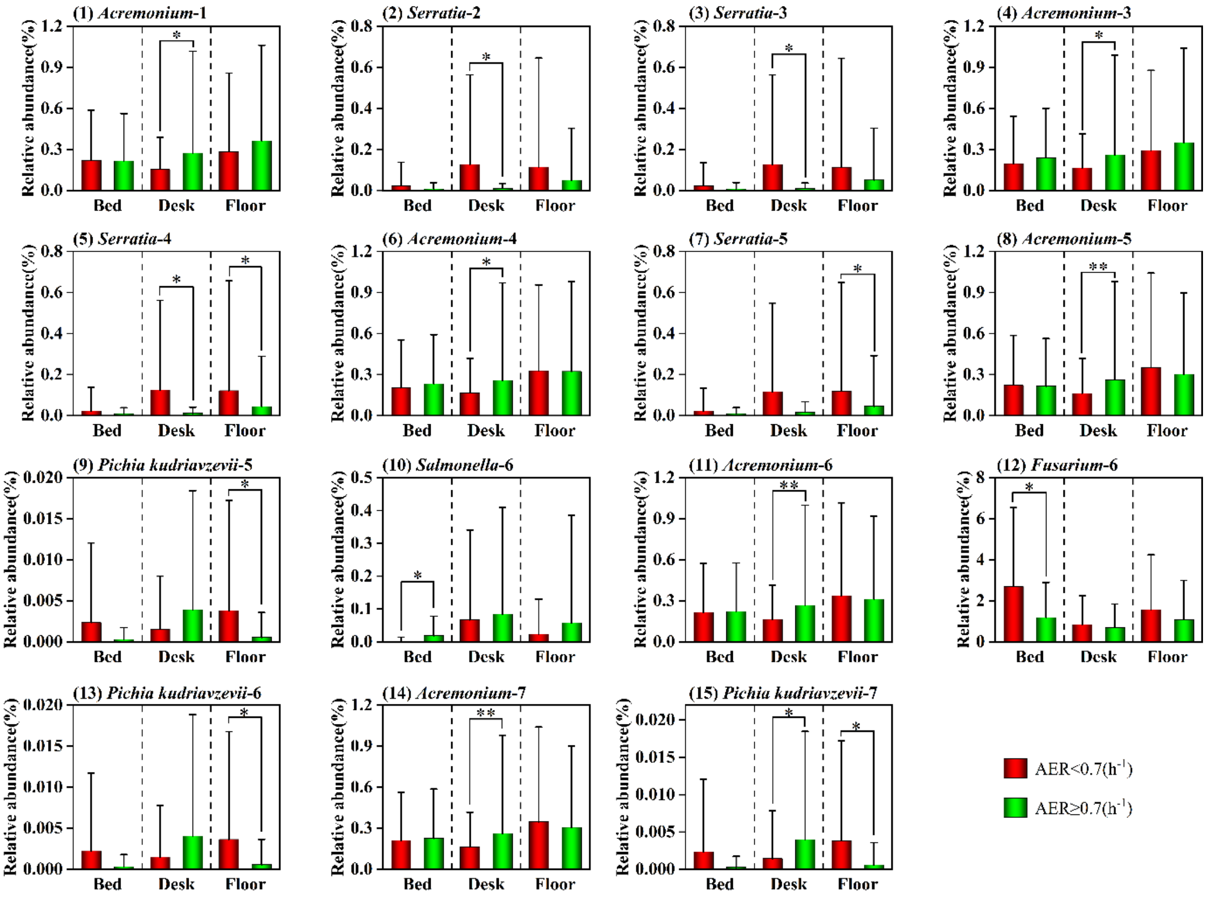

Figure S3. Differential analysis of relative abundances of additional pathogenic bacteria and fungi at different sampling sites (bed, desk, floor) between air exchange rate (AER) groups (< 0.7 h-1 vs. ≥ 0.7 h-1). The number suffix after each microbial taxon (e.g., "_1" to "_7") indicates the day prior to sampling for which the AER was assessed. * $P < 0.05$, ** $P < 0.01$, *** $P < 0.001$.
